# Excess Respiratory Hospitalisations Associated with Influenza, Respiratory Syncytial Virus and SARS‐CoV‐2 in Singapore from 2015 to 2023

**DOI:** 10.1111/irv.70098

**Published:** 2025-04-08

**Authors:** Chia Hui Qi, Robyn Lim, Rachael Pung

**Affiliations:** ^1^ Ministry of Health Singapore; ^2^ Centre for the Mathematical Modelling of infectious Diseases, Centre for Epidemic Prepardness and Response, and Department of Infectious Disease Epidemiology London School of Hygiene and Tropical Medicine London UK

**Keywords:** hospitalisation, influenza, respiratory, RSV, SARS‐CoV‐2, Singapore

## Abstract

**Background:**

The patterns of circulation and burden of influenza and respiratory syncytial virus (RSV) in Singapore are affected by the COVID‐19 pandemic containment measures. These patterns in relation to SARS‐CoV‐2 in a post‐pandemic era are unclear.

**Methods:**

Using data from 2015 to 2023, we estimated excess influenza‐, RSV‐ and SARS‐CoV‐2‐associated hospitalisation in Singapore, adjusted for rhinovirus/enterovirus activity in generalised additive models. The data include pneumonia and influenza (P&I) hospitalisation from a national inpatient database and a community‐wide acute respiratory infection (ARI) sentinel surveillance programme, stratified by age groups.

**Results:**

Across all age groups, the proportion of hospitalisation associated with influenza, SARS‐CoV‐2 and RSV was 13.2% (95% CI 5.0%–21.6%), 19.3% (95% CI 13.8%–25.0%) and 4.0% (95% CI 0.9%–12.1%) in 2023, respectively. From 2019 to 2023, all‐age influenza‐associated hospitalisation declined from 264.4 per 100,000 person‐years (95% CI 214.2–313.2) to 203.7 per 100,000 person‐years (95% CI 76.8–333.6). In contrast, all‐age RSV‐associated hospitalisation after the pandemic was 62.2 per 100,000 person‐years (95% CI 13.8–186.9), similar to pre‐pandemic observations. Peak seasonal influenza occurred 3–8 weeks later as compared with the time of pre‐pandemic peak influenza activity.

**Conclusion:**

The overall burden of influenza has declined after the COVID‐19 pandemic and its burden is comparable with SARS‐CoV‐2. Furthermore, shifts in the timing of peak influenza activity suggest a potential need to review the timing of vaccine recommendations in Singapore.

## 1 Introduction

With the emergence of SARS‐CoV‐2 in 2019, population‐wide public health and social measures (PHSMs) were implemented to contain the COVID‐19 pandemic. This has changed the circulation patterns of other respiratory pathogens in terms of the overall and age‐specific burden of disease and the time of seasonal outbreaks [1, 2, 3, 4]. Specifically, the global incidence of influenza and respiratory syncytial virus (RSV) fell by more than 60% from 2019 to 2021 based on a systematic review study [1]. For children aged below 5 years, the burden of RSV‐associated hospitalisation decreased by 80% in high‐income countries in 2020 with signs of recovery to pre‐pandemic levels by 2022 [2]. Annual peaks for influenza outbreaks have been reported to occur 2 months earlier than the pre‐pandemic period in multiple countries and outbreak seasons [4].

However, these multi‐country studies compared pre‐ and post‐COVID‐19 patterns of respiratory virus circulation span across 2020–2022 [1, 2, 4] when the COVID‐19 outbreak was ongoing. As such, study results were influenced by ongoing PHSMs, voluntary changes in social behaviour or both [5, 6]. Thus, it is unclear whether the observed circulation patterns of seasonal respiratory viruses from 2020 to 2022 will continue. Understanding the changes in the circulation patterns of seasonal respiratory viruses is crucial as it affects the cost‐effectiveness of existing and new vaccines. Furthermore, the shift in outbreak periods would influence the optimal time to promote vaccination prior to a seasonal outbreak.

Located near the equator, Singapore has a population of about 5.9 million inhabitants [7]. The country experiences year‐round circulation of influenza and RSV amongst many other respiratory viruses and bacteria. During the COVID‐19 pandemic, there was ongoing surveillance for these respiratory pathogens. Using data from the community Acute Respiratory Infection (ARI) and Influenza‐like Illness (ILI) surveillance programme and reported pneumonia and influenza (P&I) hospitalisations, we estimated the changes in the burden of influenza‐, RSV‐ and SARS‐CoV‐2‐associated P&I hospitalisation from 2015 to 2023.

## 2 Methods

### 2.1 Hospitalisation Records

We obtained hospitalisation records from January 2015 to December 2023 of patients with P&I, upper respiratory tract infections (URTI) and lower respiratory tract infections (LRTI) with a principal diagnosis of ICD10 J09‐J18, J06 and J20‐22, respectively, from the national inpatient database. Data from January 2015 to December 2019 were used to establish the 5‐year baseline non‐pandemic burden of influenza‐ and RSV‐associated hospitalisation. Individual hospitalisation records were aggregated to weekly counts by admission dates and stratified into the following age groups: < 1 year, 1–4 years, 5–9 years, 10–19 years, 20–59 years, ≥ 60 years and ≥ 65 years for age‐specific analysis.

### 2.2 Community Virological Surveillance

The Ministry of Health, Singapore, has a community‐wide sentinel ARI surveillance programme to monitor the trends of circulating viruses in Singapore. Twenty‐five government and more than 180 private primary care clinics, providing 30% of all primary health services to the population, were enrolled into the programme. Consent was obtained for the collection of respiratory samples from patients with ARI symptoms when they sought outpatient consultation at one of the sentinel primary care clinics. These samples were routinely submitted to the National Public Health Laboratory (NPHL) and tested using the FilmArray Respiratory Panel and/or real‐time reverse transcription‐polymerase chain reaction (RT‐PCR) for a range of respiratory pathogens. We obtained the weekly positivity rates for influenza, RSV and rhinoviruses/enteroviruses, and derived from the proportion of samples testing positive for the viruses from 2015 to 2023 as a proxy for viral activity in the community. These viruses were the top three common circulating viruses in the community, accounting for more than 60% of the respiratory samples with a positive outcome.

From 2020 to 2021, there was comprehensive testing and mandatory notification of COVID‐19 cases in Singapore [8]. As such, locally reported COVID‐19 cases from 2020 to 2021 were a reliable proxy for SARS‐CoV‐2 viral activity. As Singapore progressively reduced the requirements for testing and notification of COVID‐19 cases from 2022 onwards [9, 10], we used the weekly positivity rates for SARS‐CoV‐2 obtained from the ARI sentinel surveillance programme to estimate the level of SARS‐CoV‐2 activity from 2022 to 2023.

We obtained the weekly ARI attendance in government primary care clinics and calculated the average daily attendance each week to account for reduced attendance during public holidays. We multiplied the weekly viral positivity rates and the weekly average daily ARI attendance to estimate the weekly average daily incidence of influenza, RSV, SARS‐CoV‐2 and rhinovirus/enterovirus in government primary care clinics. All‐age positivity of respiratory pathogens, ARI attendance and reported COVID‐19 cases were used to model the age‐specific hospitalisation outcomes given the small number of respiratory samples in age‐specific groups.

## 3 Statistical Analysis

We used a generalised additive negative binomial model to model weekly influenza‐, RSV‐ and SARS‐CoV‐2‐associated P&I, URTI and LRTI hospitalisation rates from 2015 to 2023, adjusted for rhinoviruses/enteroviruses as a confounder. Generalised additive models (GAMs) have been used in several hospitalisation burden studies and have the advantage of accounting for non‐linear effects using smoothing functions [11, 12, 13, 14, 15]. Data on the COVID‐19 pandemic from 2020 to the World Health Organization's declaration of the end of COVID‐19 as a global health emergency on 5 May 2023 were included in the analysis. We also modelled the weekly influenza‐, RSV‐ and SARS‐CoV‐2‐associated P&I, LRTI and URTI hospitalisation, respectively, and presented the findings in the Supporting Information. This allows our study findings to be compared with other studies reporting influenza‐associated P&I hospitalisations. To control for non‐linear seasonality trends over time, a smoothing spline function was incorporated into the model with the full model defined as follows:

$$\mathrm{Log}\left[E\left(\text{weekly admissions}\right)\right]=s\left(t\right)+\sum_{v}s\left(\text{weekly incidence of}\;v\right)$$

where s refers to a smoothing spline function, t refers to the week index (1 to end of time period) and v∈ {influenza, RSV, SARS‐CoV‐2, rhinovirus/enterovirus}. We modelled the weeks and incidence variables using thin plate regression splines [16]. The degree of smoothness was selected automatically using restricted maximum likelihood to balance model fit and complexity. All‐age and age‐specific models were fitted to estimate the hospitalisations. To account for the changes in the SARS‐CoV‐2 incidence proxy variable and fluctuations in influenza viral activity, we stratified the analysis into four time periods determined a priori (Table S1).

We defined the excess hospitalisations associated with a virus as the difference between the estimated hospitalisations and the counterfactual hospitalisations when the positivity of the virus was zero. Negative excess hospitalisation is not meaningful in this context as it implies a protective effect. Hence, negative estimates were set to zero [13, 14, 15]. We estimated the annual hospitalisation excess incidence rates (per 100,000 person‐years) by dividing the model derived excess rates with the mid‐year total population estimates. The proportion of excess hospitalisations was estimated by dividing the total number of excess hospitalisations by the total number of observed hospitalisations. We used the standard error of the mean to establish the 95% confidence intervals (95% CI) for all metrics. All statistical analysis was conducted using R, version 3.6.3.

## 4 Results

### 4.1 Viral Surveillance

**FIGURE 1:**
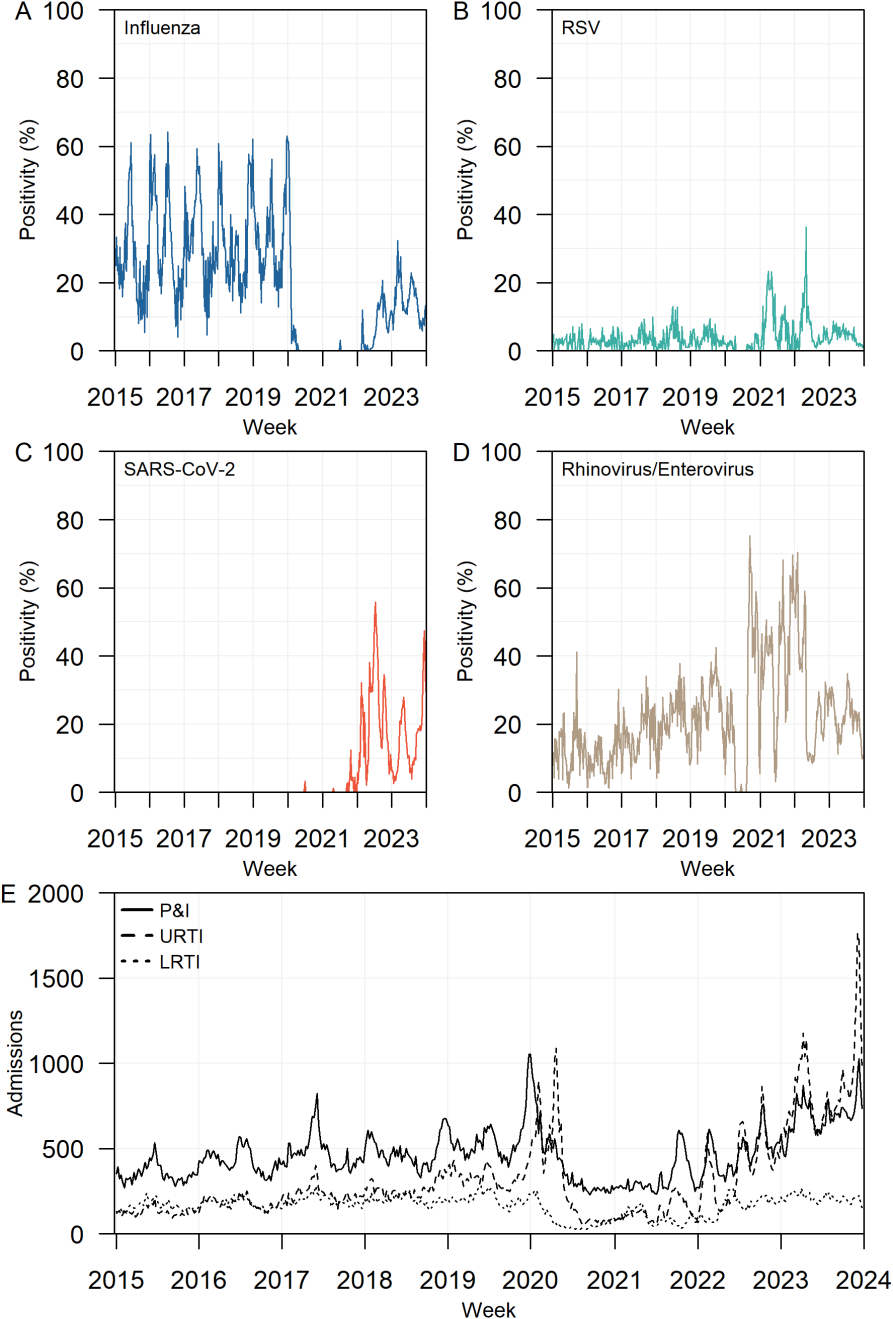
Weekly viral and respiratory infection surveillance from 2015–2023. Weekly proportions of respiratory samples from the sentinel ARI surveillance programme tested positive for (A) influenza, (B) RSV, (C) SARS‐CoV‐2 and (D) rhinovirus/enterovirus. Weekly number of hospital admissions by diagnosis codes (E). Horizontal axis labels represent the first e‐week of each year.

Prior to the COVID‐19 pandemic, we observed bimodal peaks in influenza viral activity from 2015 to 2019, with peak influenza activity occurring around May to July and December to February (Figure 1A). These peak periods correspond to the Southern and Northern Hemisphere winter influenza outbreak seasons respectively. No obvious seasonal fluctuations were observed for RSV and rhinovirus/enterovirus (Figure 1B,D). During the COVID‐19 pandemic, we observed a disruption to the viral circulation patterns in the community. Influenza positivity rates plummeted to near zero in 2020 and 2021, before increasing in mid‐2022 but peak positivity rates were at most halved of the pre‐pandemic rates (Figure 1A). Similarly, RSV and rhinovirus/enterovirus positivity declined to near zero in 2020 but rebounded in 2021 with peak positivity rates exceeding twice their pre‐pandemic rate (Figure 1B,D). Peak influenza activity in 2022 and 2023 occurred around August to September for the Southern Hemisphere winter and February and March for the Northern Hemisphere winter.

In general, SARS‐CoV‐2 positivity rates fluctuated in tandem with the reported COVID‐19 case notifications (Figure S1). From 2020 to 2021, separate testing and notification guidelines were in place for COVID‐19 cases and the sentinel testing of SARS‐CoV‐2 increased from 2022 onwards (Figures 1C and S1). Peak SARS‐CoV‐2 positivity was reported with peaks occurring in July 2022 and April 2023 corresponding to the Omicron BA.4/5 and XBB.1.16 outbreak in Singapore (Figure 1c).

### 4.2 Reported and Excess Hospitalisations

The average weekly P&I, URTI and LRTI hospitalisations from 2015 to 2023 were 464, 315 and 166, respectively (Figure 1E and Tables S2–S4). A spike in URTI hospitalisations was observed in early 2020, corresponding to the increase in COVID‐19 suspect and confirmed cases (Figure 1E).

### 4.3 Influenza

We estimated an increase in all‐age excess influenza‐associated combined P&I, URTI and LRTI hospitalisation rates from 115.1 (95% CI 87.4–144.1) per 100,000 person‐years to 264.4 (95% CI 214.2–313.2) per 100,000 person‐years from 2015 to 2019 (Table 1). This declined to 74.0 (95% CI 29.3–128.3) per 100,000 person‐years in 2020 and 22.1 (95% CI 6.6–52.4) per 100,000 person‐years in 2021. However, the excess influenza‐associated hospitalisation rate rebounded to 47.8 (95% CI 12.4–117.4) per 100,000 person‐years in 2022 and 203.7 (95% CI 76.8–333.6) per 100,000 person‐years in 2023. These trends were also observed among age‐specific excess influenza‐associated hospitalisation.

**TABLE 1:** Estimated mean yearly age‐stratified influenza‐associated excess P&I, URTI and LRTI hospitalisation rates (per 100,000 person‐years) from 2015 to 2023. Ninety‐five percent CI are in brackets.

Year	Age groups							
	All	< 1	1–4	5–9	10–19	20–59	≥ 60	≥ 65
2015	115.1 (87.4–144.1)	2321.6 (1538.9–3116.3)	824.8 (539.7–1103.3)	189.7 (133.2–248.5)	63.5 (41.3–83.8)	22.2 (12.6–32.6)	366.5 (273.8–463.4)	503.0 (370.7–641.8)
2016	178.5 (146.3–210.3)	3088.7 (2047.2–4093.2)	1142.6 (777.1–1487.1)	309.0 (242.9–374.4)	117.5 (87.4–144.2)	38.6 (26.2–52.3)	497.9 (400.5–597)	665.6 (526.6–804.5)
2017	218.3 (178.8–256.8)	3384.6 (2193.4–4534.9)	1327.2 (932.0–1705.9)	297.6 (229.5–362.3)	118.5 (88.7–144.1)	47.7 (33.6–63.3)	627.7 (504.5–752.0)	847.5 (675.3–1021.3)
2018	226.4 (181.8–269.1)	3278.2 (2080.4–4499.4)	1409.5 (956.7–1823.3)	322.4 (249.8–394.0)	111.4 (82.4–136.8)	48.3 (33.6–63.7)	660.0 (518.0–797.8)	864.2 (671.4–1050.9)
2019	264.4 (214.2–313.2)	3835.9 (2531.0–5084.5)	1692.7 (1205.7–2150.2)	415.7 (324.6–504.5)	149.3 (110.0–182.5)	52.7 (36.9–70.3)	744.7 (574.8–908.7)	947.4 (724.4–1162.2)
2020	74.0 (29.3–128.3)	1088.5 (602.1–1587.9)	379.4 (167.2–625.0)	144.3 (74.7–221.3)	51.3 (26.0–80.2)	45.1 (9.8–90.6)	211.0 (100.4–344.5)	259.5 (129.9–420.8)
2021	22.1 (6.6–52.4)	558.6 (187.2–1164.6)	180.7 (48.2–388.3)	46.0 (14.6–83.2)	18.5 (6.1–32.5)	9.1 (2.5–24.4)	83.6 (30.9–168.6)	103.1 (35.8–213.2)
2022	47.8 (12.4–117.4)	672.9 (110.7–2054.2)	226.4 (41.4–549.2)	168.6 (70.6–291.8)	55.7 (30.1–88.6)	18.0 (4.9–39.0)	186.0 (44.7–473.5)	704.8 (297.7–1138.0)
2023	203.7 (76.8–333.6)	2190.8 (614.4–4132.4)	901.3 (347.5–1514.8)	417.2 (157.1–699.8)	133.4 (73.1–190.6)	44.8 (15.2–77.7)	852.0 (360.8–1374.2)	991.6 (429.9–1617.9)

Mean excess influenza‐associated combined P&I, URTI and LRTI hospitalisation rates were 5.2–6.9 times higher among infants aged < 1 year as compared with the elderly aged ≥ 60 years from 2015 to 2021 (Table 1). This observation was reduced in 2022 and 2023 where the estimated mean excess influenza‐associated hospitalisation rates were 2.5–3.6 times higher among the infants aged < 1 year compared with the elderly aged ≥ 60 years. Regardless, the hospitalisation rates in both infants aged < 1 year and elderly aged ≥ 60 years were 10 times higher than those aged 10–59 years and 2 times higher than those aged 1–4 years. However, we estimated that the mean P&I only hospitalisation burden for influenza was 1.2 times higher in young children aged 1–4 years as compared with infants aged < 1 year in 2019 and this doubled to 2 times in 2023 (Table S5). Using P&I hospitalisation only, the all‐age influenza‐associated is about 2–3 times lower than the main analysis (Tables S5–S7). The proportion of all‐age P&I, URTI and LRTI hospitalisations attributable to influenza was 19.0% (95% CI 14.4%–23.7%) to 26.6% (95% CI 21.6%–31.5%) from 2015 to 2019 but decreased to 9.9% (95% CI 3.9%–17.1%) in 2020 before increasing to 13.2% (95% CI 5.0%–21.6%) in 2023 (Table S8).

### 4.4 RSV

We estimated that the all‐age excess RSV‐associated combined P&I, URTI and LRTI hospitalisation rates nearly doubled from 36.0 (95% CI 22.6–53.8) per 100,000 person‐years in 2015 to 67.0 (95% CI 41.7–100.8) per 100,000 person‐years in 2019 (Table 2). Specifically, the mean excess hospitalisation rates in 2019 were at least 1.6 times higher the rates in 2015 for age groups spanning < 1–59 years. All‐age excess RSV‐associated hospitalisation rates were halved to 32.6 (95% CI 6.9–86.1) per 100,000 person‐years in 2022 before increasing back to 62.2 (95% CI 13.8–186.9) per 100,000 person‐years in 2023.

**TABLE 2:** Estimated mean yearly age‐stratified RSV‐associated excess P&I, URTI and LRTI hospitalisation rates (per 100,000 person‐years) from 2015 to 2023; Ninety‐five percent CI are in brackets.

Year	Age groups							
	All	< 1	1–4	5–9	10–19	20–59	≥ 60	≥ 65
2015	36.0 (22.6–53.8)	1208.6 (759.2–1693.7)	297.3 (168.3–443.6)	91.9 (52.7–138.2)	26.0 (14.8–40.0)	11.0 (6.5–16.9)	156.9 (98.2–228.4)	216.0 (136.6–317.7)
2016	44.0 (27.5–63.9)	1469.2 (973.3–2050.9)	320.6 (195.9–487.1)	126.9 (83.5–177.8)	42.3 (27.0–60.7)	13.2 (7.9–20.0)	160.4 (103.3–238.1)	216.8 (138.5–319.0)
2017	58.9 (38.7–84.7)	1539.8 (961.4–2203.0)	377.7 (225.0–570.1)	110.0 (68.2–163.5)	42.1 (25.6–61.6)	16.3 (10.5–24.4)	222.5 (148.0–316.2)	295.8 (196.8–427.8)
2018	52.1 (32.1–78.1)	1754.6 (1201.9–2395.5)	449.0 (282.5–656.0)	111.5 (63.8–170.4)	43.2 (26.9–61.1)	14.6 (8.3–22.9)	180.0 (104.2–281.3)	238.4 (136.2–371.5)
2019	67.0 (41.7–100.8)	2026.9 (1348.6–2814.0)	535.5 (331.0–792.1)	160.0 (89.8–242.2)	56.3 (31.6–83.0)	17.6 (10.1–27.5)	190.3 (104.1–313.7)	249.5 (132.4–416.4)
2020	51.2 (17.8–100.0)	656.7 (261.3–1166.7)	194.8 (68.4–413.1)	71.0 (27.6–145.5)	42.7 (17.7–74.7)	45.9 (9.9–90.6)	128.5 (44.3–258.6)	158.5 (60.6–316.1)
2021	39.8 (12.7–80.4)	1391.3 (639.6–2137.9)	343.9 (99.3–623.2)	38.7 (7.9–82.4)	22.0 (7.4–37.1)	13.9 (3.7–31.7)	114.4 (42.1–221.4)	145.5 (53.7–282.4)
2022	32.6 (6.9–86.1)	773.1 (135.7–2071.2)	158.1 (20.7–634.1)	66.8 (6.1–164.8)	21.2 (3.9–47.8)	7.7 (1.3–22.7)	176.5 (34.1–437.2)	161.6 (24.7–494.6)
2023	62.2 (13.8–186.9)	1520.3 (347.7–3333.1)	646.9 (103.8–1386.1)	192.2 (34.7–447.9)	69.1 (24.2–131.8)	11.7 (1.6–39.5)	226.3 (30.8–797.4)	268.0 (41.8–926.9)

The mean excess RSV‐associated combined P&I, URTI and LRTI hospitalisation rate was generally the highest for infants aged < 1 year, more than five times higher than the elderly ≥ 65 years before 2020. Similar trends were observed in 2023, post‐COVID‐19 pandemic. However, based on P&I hospitalisation only, we estimated that the mean burden for RSV in infants aged < 1 year was two times higher than the elderly ≥ 65 years before 2020 (Table S9). Using P&I hospitalisation only, the all‐age RSV‐associated is about two to three times lower than the main analysis (Tables S9–S11). All‐age P&I, URTI and LRTI hospitalisations attributable to RSV ranged from 5.9% to 6.7% from 2015 to 2019 and decreased to 3.5% (95% CI 0.7%–9.1%) in 2022 and is steadily to 4.0% (95% CI 0.9%–12.8%) in 2023 with CIs overlapping with pre‐pandemic excess proportion estimates (Table S12).

### 4.5 SARS‐CoV‐2

All‐age excess SARS‐CoV‐2‐associated combined P&I, URTI and LRTI hospitalisation rates increased by about 1.5 times from 117.8 (95% CI 84.3–165.6) per 100,000 person‐years in 2020 to 191.3 (95% CI 127.9–262.0) per 100,000 person‐years in 2022 and continued to increase to 298.2 (95% CI 212.9–386.1) in 2023 (Table 3). For the elderly aged ≥ 60 years, excess SARS‐CoV‐2 hospitalisation rates increased about threefold from 2020 to 2021.

**TABLE 3:** Estimated mean yearly age‐stratified SARS‐CoV‐2‐associated excess P&I, URTI and LRTI hospitalisation rates (per 100,000 person‐years) from 2020 to 2023. Ninety‐five percent CI are in brackets.

Year	Age groups							
	All	< 1	1–4	5–9	10–19	20–59	≥ 60	≥ 65
2020	117.8 (84.3–165.6)	450.7 (165.3–947)	168.5 (63.5–372.9)	78.0 (29.5–146.8)	35.0 (15.7–65.3)	130.8 (94.4–168.4)	149.7 (67.5–286)	190.8 (93.6–358.2)
2021	105.9 (81.8–134.1)	482.8 (167.9–1104.2)	140.9 (37.2–324.2)	34.7 (8.3–73.2)	16.3 (4.2–30.6)	41.2 (29.5–53.3)	346.9 (265.0–440.3)	438.6 (338.4–557.6)
2022	191.3 (127.9–262.0)	587.6 (93.7–1957.4)	99.5 (13.7–576.4)	76.4 (14.4–198.7)	40.5 (12.7–74.7)	25.6 (7.7–45.0)	970.3 (665.9–1255.9)	1310.5 (950.8–1659.6)
2023	298.2 (212.9–386.1)	1590.7 (527.9–3057.7)	320.4 (67.7–833.5)	189.2 (40.3–411.2)	34.8 (10.6–78.5)	46.2 (25.7–67.8)	1542.2 (1144.7–1926.4)	1947.6 (1464.8–2418.1)

The fluctuations in SARS‐CoV‐2‐associated P&I hospitalisations were different from URTI hospitalisations. SARS‐CoV‐2‐associated all‐age P&I hospitalisation increased by four times from 2020 to 2023 with a consistent increase over each year (Table S13). However, we estimated that SARS‐CoV‐2‐associated URTI hospitalisation decreased from 93.0 (95% CI 72.3–118.8) per 100,000 person‐years in 2020 to 47.4 (95% CI 36.6–58.8) per 100,000 person‐years in 2021 before increasing to 105.6 (95% CI 73.8–135.3) per 100,000 person‐years in 2022 and 191.5 (95% CI 147.1–234.2) per 100,000 person‐years in 2023 (Table S14). Using P&I hospitalisation only, the all‐age SARS‐CoV‐2‐associated is about two to five times lower than the main analysis (Tables S13–S15). The proportion of all‐age P&I, URTI and LRTI hospitalisations attributable to SARS‐CoV‐2 was 15.7% (95% CI 11.3%–22.1%) in 2020 and remains similar at 19.3% (95% CI 13.8%–25.0%) in 2023 (Table S16).

## 5 Discussion

Using the community virological surveillance data and P&I, URTI and LRTI hospitalisation data, we modelled the excess influenza‐, RSV‐ and SARS‐CoV‐2‐associated hospitalisation from 2015 to 2023 in Singapore. We showed that the excess hospitalisation burden for influenza was four times higher than RSV before the COVID‐19 pandemic. Post‐COVID‐19 pandemic, the burden of influenza‐ and SARS‐CoV‐2‐associated combined P&I, URTI and LRTI hospitalisation was comparable and were 3.3 times and 4.8 times higher than RSV. Taking into consideration all P&I, URTI and LRTI hospitalisations, influenza and SARS‐CoV‐2 both accounted more than 30% of the hospitalisations post‐COVID‐19 pandemic and RSV accounted for less than 10%. Our study highlights the changes in the burden of circulating respiratory viruses with the introduction of SARS‐CoV‐2, and this helps to inform the extent of public health measures required in the post‐COVID‐19 landscape in Singapore.

Our findings in the community virological surveillance of influenza and SARS‐CoV‐2 partially corroborate with the observations in some countries. Peak influenza positivity in the 2023 winter season was 30%–40% lower than that in the pre‐COVID‐19 pandemic years for countries such as the United States of America, the United Kingdom and other European countries, Australia and Hong Kong [17, 18, 19, 20, 21]. In Singapore, we also observed a reduction in peak influenza positivity in 2023 by more than 50% as compared with pre‐pandemic years. Across most temperate countries, the period of peak influenza activity in 2023 was similar to pre‐COVID‐19 observations [17, 18, 19, 20]. In contrast, Singapore is in the equatorial region and experiences year‐round circulation of influenza. The time of peak seasonal influenza viral activity in the community in 2023 was about 8 and 3 weeks later for the Northern and Southern Hemisphere winter seasons, respectively, when compared with pre‐pandemic years. Preliminary data in 2024 suggest that this difference was reduced to about 1–3 weeks. Based on observed data alone, peak influenza activity corresponding to the Southern Hemisphere winter occurred 10–15 weeks after peak SARS‐CoV‐2 activity in 2022 and 2023. Changes in population behaviour, such as mask‐wearing or self‐isolation, during SARS‐CoV‐2 outbreak waves in the post‐COVID‐19 pandemic era could lower and delay the onset of other seasonal virus activity [22, 23]. As such, the optimal timing of influenza vaccination campaigns could potentially be dependent on the outbreak cycles of SARS‐CoV‐2 and further observations are required.

Given the reduction in influenza positivity in the community, we estimated a corresponding 1.3 times reduction in the mean excess all‐age influenza‐associated P&I, URTI and LRTI hospitalisation as compared with pre‐COVID‐19 pandemic years. When comparing across age groups, the mean influenza‐associated hospitalisations among those aged < 1 year and ≥ 60 years remained at higher risk as compared with those aged 10 to 59. These findings corroborate with other high‐income countries [24]. In Singapore, the influenza‐associated P&I hospitalisation in infants aged < 1 year was two times higher than in young children aged 1–4 years in 2023. Prior to the pandemic, both age groups had comparable influenza‐associated P&I hospitalisation burden. The reduced influenza circulation during the COVID‐19 pandemic years in 2020–2021 potentially resulted in lowered exposure and hence risk of infection to influenza. This potentially creates a temporary immunity debt [25], thereby increasing the age of influenza infection. Modelling studies in other countries for other respiratory diseases such as RSV, in addition to influenza, mirrored our observations [26, 27, 28]. However, this observation is not expected to persist [27, 28] and current influenza vaccination recommendations remain relevant for those aged 6 months to 5 years and 65 years and above [29]. Continued surveillance is required to better understand the changes in age distribution among influenza hospitalisations.

All‐age and age‐specific RSV‐associated hospitalisation remains low and was about two to four times lower than the hospitalisation burden reported in a multi‐country systematic review and meta‐analysis [30]. Unlike most temperate countries which exhibit seasonal fluctuations in RSV transmission [31, 32, 33], viral circulation patterns in Singapore did not exhibit seasonal peaks in the years before 2020 and from 2022 to 2023. In Singapore, the burden of RSV‐associated P&I hospitalisation in infants < 1 year is two times higher as compared with elderly aged ≥ 60 years. In other developed countries, the burden of RSV hospitalisation in young children aged below 5 years was about two to three times the findings in Singapore [1, 30] while the hospitalisation rate in elderly ≥ 65 years was 245 per 100,000 person‐years [30], comparable with the findings in Singapore. Studies have shown that RSV‐vaccinated elderly aged ≥ 60 years had over 80% reduction in the risk of RSV‐associated lower respiratory tract disease compared with those who were not vaccinated [34, 35]. For Singapore, further studies on the cost‐effectiveness of RSV vaccines for those aged < 5 and ≥ 60 years should be conducted to review the suitability of inclusion in the national childhood and adult immunisation schedules given that the overall burden from influenza and SARS‐CoV‐2 is much higher than RSV.

Our study has a few strengths and limitations. First, we accounted for rhinovirus/enterovirus circulation activity as a confounder in our statistical analysis. It was important as rhinovirus/enterovirus accounted for over 20% of the sentinel respiratory samples with a positive outcome. Second, we investigated the use of SARS‐CoV‐2 virological surveillance 2 years after the start of the COVID‐19 pandemic to establish SARS‐CoV‐2‐associated P&I hospitalisation. As Singapore progressively reduced testing of COVID‐19, the COVID‐19 notifications would decrease over time and reliance on these notifications would underestimate the burden of SARS‐CoV‐2 hospitalisations. Third, our modelling framework collectively estimated the hospitalisation burden of influenza, RSV and SARS‐CoV‐2 given that all three pathogens co‐circulate in the community.

One limitation was the assumption that community viral patterns were reflective of hospitalised viral patterns. Due to the lack of viral positivity data of hospitalised patients, we had to use the community virological surveillance data as a proxy for viral activity among hospitalised patients. However, even if hospital laboratory viral positivity data were available, these outcomes in hospitalised patients are potentially affected by the time of administrating medicine and the time of testing. As such, interpretation of hospital laboratory data should be done with caution. Future severe acute respiratory illness (SARI) hospital sentinel surveillance could incorporate random sampling of SARI patients for testing. This allows us to estimate the hospitalisation burden associated with each respiratory pathogen and the surveillance could be gradually expanded to ARI admissions. Second, changes in the suspect case definition and case‐finding strategy over the pandemic from 2020 to 2022 could bias the observed hospitalisations in either direction. We included these observations over the pandemic in our study, but findings should be interpreted carefully. Third, we used COVID‐19 case notifications from 2020 to 2021 before switching to SARS‐CoV‐2 positivity from the sentinel surveillance programme in 2022 onwards. As such, we performed the model fitting over four time periods to account for the absence of SARS‐CoV‐2 before 2020, the change in COVID‐19 surveillance data and changes in viral circulation patterns. The change in COVID‐19 surveillance data was prompted by the change in testing and reporting patterns for SARS‐CoV‐2 over the COVID‐19 pandemic. In general, peak SARS‐CoV‐2 positivity data align with our reported COVID‐19 cases and would suitably represent the trend of SARS‐CoV‐2 viral activity as the case ascertainment decreases over time. Finally, patients infected with influenza could be diagnosed with circulatory diseases, and the hospitalisation burden from this group was not accounted for in this study. Accounting for the burden from this group would require further analysis of the proportion of influenza‐positive cases by principal diagnosis.

## 6 Conclusions

Our findings suggest a decrease in the hospitalisation burden of influenza but no change in the burden of RSV 4 years after the introduction of SARS‐CoV‐2. Furthermore, concurrent outbreaks of influenza and SARS‐CoV‐2 were not commonly observed, and the timing of influenza outbreaks was delayed as compared with pre‐pandemic years. Future influenza and RSV vaccine recommendations should consider the overall burden of respiratory disease in the post‐COVID‐19 pandemic era.

## Author Contributions

**Chia Hui Qi:** software, data curation, formal analysis, investigation, validation, visualization, writing – review and editing, writing – original draft. **Robyn Lim:** software, data curation, formal analysis, validation, investigation, visualization, writing – review and editing. **Rachael Pung:** conceptualization, methodology, investigation, supervision, visualization, writing – review and editing.

## Conflicts of Interest

The authors declare no conflicts of interest.

### Peer Review

The peer review history for this article is available at https://www.webofscience.com/api/gateway/wos/peer‐review/10.1111/irv.70098.

## Supporting information


**Figure S1** Weekly COVID‐19 local case notifications tracked by the Ministry of Health and SARS‐CoV‐2 positivity from the sentinel ARI surveillance programme.
**Table S1.** Time periods used for model stratification.
**Table S2.** Reported mean weekly age‐stratified P&I hospitalisations, from 2015 to 2023.
**Table S3.** Reported mean weekly age‐stratified URTI hospitalisations, from 2015 to 2023.
**Table S4.** Reported mean weekly age‐stratified LRTI hospitalisations, from 2015 to 2023.
**Table S5.** Estimated mean yearly age‐stratified influenza‐associated excess P&I hospitalisation rates (per 100,000 person‐years) from 2015–2023. 95% CI in brackets.
**Table S6.** Estimated mean yearly age‐stratified influenza‐associated excess URTI hospitalisation rates (per 100,000 person‐years) from 2015–2023. 95% CI in brackets.
**Table S7.** Estimated mean yearly age‐stratified influenza‐associated excess LRTI hospitalisation rates (per 100,000 person‐years) from 2015–2023. 95% CI in brackets.
**Table S8.** Estimated yearly age‐stratified influenza‐associated excess P&I, URTI and LRTI hospitalisation proportion from 2015–2023. 95% CI in brackets.
**Table S9.** Estimated mean yearly age‐stratified RSV‐associated excess P&I hospitalisation rates (per 100,000 person‐years) from 2015–2023. 95% CI in brackets.
**Table S10.** Estimated mean yearly age‐stratified RSV‐associated excess URTI hospitalisation rates (per 100,000 person‐years) from 2015–2023. 95% CI in brackets.
**Table S11.** Estimated mean yearly age‐stratified RSV‐associated excess LRTI hospitalisation rates (per 100,000 person‐years) from 2015–2023. 95% CI in brackets.
**Table S12.** Estimated yearly age‐stratified RSV‐associated excess P&I, URTI and LRTI hospitalisation proportion from 2015–2023. 95% CI in brackets.
**Table S13.** Estimated mean yearly age‐stratified SARS‐CoV‐2‐associated excess P&I hospitalisation rates (per 100,000 person‐years) from 2020–2023. 95% CI in brackets.
**Table S14.** Estimated mean yearly age‐stratified SARS‐CoV‐2‐associated excess URTI hospitalisation rates (per 100,000 person‐years) from 2020–2023. 95% CI in brackets.
**Table S15.** Estimated mean yearly age‐stratified SARS‐CoV‐2‐associated excess LRTI hospitalisation rates (per 100,000 person‐years) from 2020–2023. 95% CI in brackets.
**Table S16.** Estimated yearly age‐stratified SARS‐CoV‐2‐associated excess P&I, URTI and LRTI hospitalisation proportion from 2020–2023. 95% CI in brackets.

## Data Availability

All data supporting the findings of this study are available within the paper and the Supporting Information.
