# Supplementary material for: Excess Respiratory Hospitalisations Associated with Influenza, Respiratory Syncytial Virus and SARS‐CoV‐2 in Singapore from 2015 to 2023
Source: Influenza Other Respir Viruses. 2025 Apr 8;19(4):e70098. doi: 10.1111/irv.70098 (PMC11976161; doi:10.1111/irv.70098)
Supplement: Supplementary file 1 — Figure S1 Weekly COVID‐19 local case notifications tracked by the Ministry of Health and SARS‐CoV‐2 positivity from the sentinel ARI surveillance programme. Table S1. Time periods used for model stratification. Table S2. Reported mean weekly age‐stratified P&I hospitalisations, from 2015 to 2023. Table S3. Reported mean weekly age‐stratified URTI hospitalisations, from 2015 to 2023. Table S4. Reported mean weekly age‐stratified LRTI hospitalisations, from 2015 to 2023. Table S5. Estimated mean yearly age‐stratified influenza‐associated excess P&I hospitalisation rates (per 100,000 person‐years) from 2015–2023. 95% CI in brackets. Table S6. Estimated mean yearly age‐stratified influenza‐associated excess URTI hospitalisation rates (per 100,000 person‐years) from 2015–2023. 95% CI in brackets. Table S7. Estimated mean yearly age‐stratified influenza‐associated excess LRTI hospitalisation rates (per 100,000 person‐years) from 2015–2023. 95% CI in brackets. Table S8. Estimated yearly age‐stratified influenza‐associated excess P&I, URTI and LRTI hospitalisation proportion from 2015–2023. 95% CI in brackets. Table S9. Estimated mean yearly age‐stratified RSV‐associated excess P&I hospitalisation rates (per 100,000 person‐years) from 2015–2023. 95% CI in brackets. Table S10. Estimated mean yearly age‐stratified RSV‐associated excess URTI hospitalisation rates (per 100,000 person‐years) from 2015–2023. 95% CI in brackets. Table S11. Estimated mean yearly age‐stratified RSV‐associated excess LRTI hospitalisation rates (per 100,000 person‐years) from 2015–2023. 95% CI in brackets. Table S12. Estimated yearly age‐stratified RSV‐associated excess P&I, URTI and LRTI hospitalisation proportion from 2015–2023. 95% CI in brackets. Table S13. Estimated mean yearly age‐stratified SARS‐CoV‐2‐associated excess P&I hospitalisation rates (per 100,000 person‐years) from 2020–2023. 95% CI in brackets. Table S14. Estimated mean yearly age‐stratified SARS‐CoV‐2‐associated excess URTI hospita [file IRV-19-e70098-s001.docx]

**Supplementary Information**

**Supplementary Figure 1.** Weekly COVID-19 local case notifications tracked by the Ministry of Health and SARS-CoV-2 positivity from the sentinel ARI surveillance programme


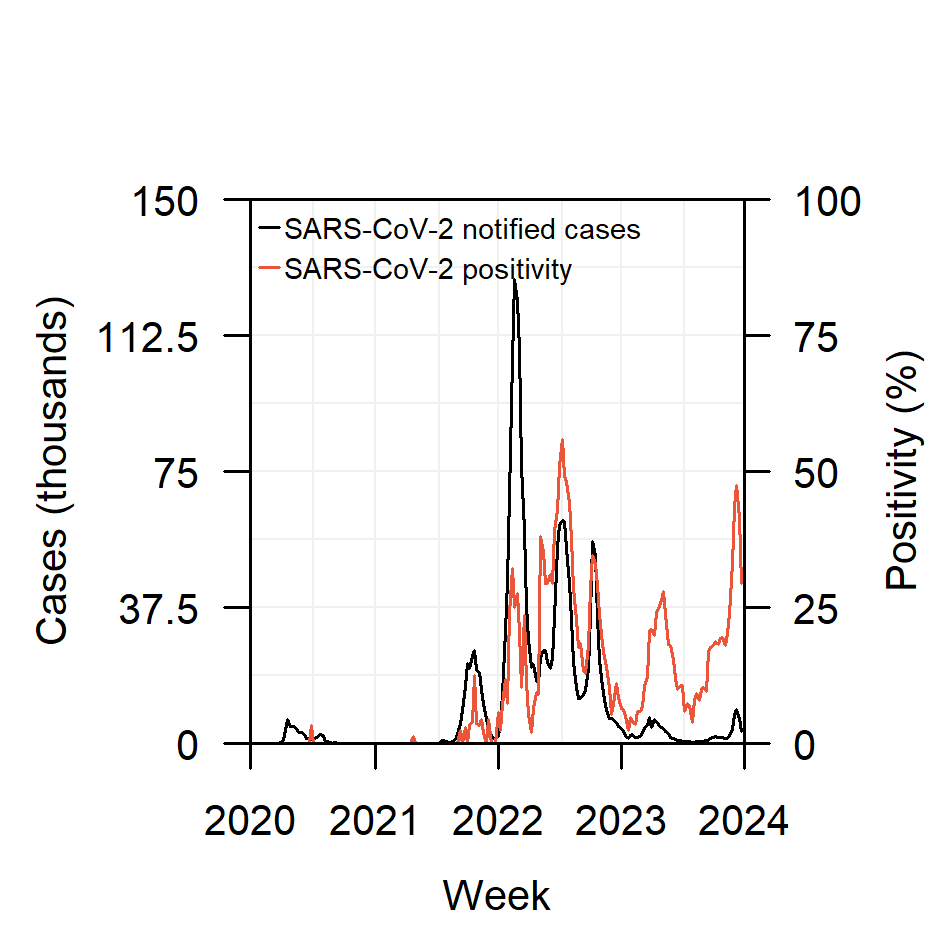


**Supplementary Table 1.** Time periods used for model stratification

| Time Period | Remarks |
| --- | --- |
| Jan 2015 – Dec 2019 | Pre-COVID-19 pandemic and analysis does not include SARS-CoV-2 associated excess hospitalisation. |
| Jan 2020 – Dec 2021 | COVID-19 notifications were mandatory and these notification served as a proxy for SARS-CoV-2 viral activity. |
| Jan 2022 – Dec 2022 | COVID-19 testing and notifications were progressively reduced hence community virological surveillance was used for monitoring SARS-CoV-2 viral activity. Community virological surveillance showed low influenza viral activity at the start of 2022. |
| Jan 2023 – Dec 2023 | Community virological surveillance used to monitor SARS-CoV-2 activity viral activity and also showed increased influenza viral activity relative to 2020-2022. |

**Supplementary Table 2.** Reported mean weekly age-stratified P&I hospitalisations, from 2015 to 2023

| Year | Age groups | | | | | | | |
| --- | --- | --- | --- | --- | --- | --- | --- | --- |
|  | All | <1 | 1 – 4 | 5 – 9 | 10 – 19 | 20 – 59 | ≥60 | ≥65 |
| 2015 | 353 | 9 | 28 | 16 | 9 | 63 | 229 | 206 |
| 2016 | 427 | 8 | 36 | 18 | 13 | 82 | 270 | 243 |
| 2017 | 476 | 11 | 41 | 16 | 10 | 87 | 311 | 279 |
| 2018 | 479 | 10 | 43 | 18 | 11 | 86 | 312 | 280 |
| 2019 | 548 | 12 | 58 | 29 | 16 | 101 | 332 | 297 |
| 2020 | 406 | 5 | 16 | 7 | 5 | 93 | 280 | 251 |
| 2021 | 329 | 3 | 6 | 1 | 1 | 50 | 270 | 245 |
| 2022 | 469 | 5 | 24 | 7 | 5 | 53 | 375 | 346 |
| 2023 | 693 | 9 | 47 | 28 | 14 | 94 | 501 | 461 |

**Supplementary Table 3.** Reported mean weekly age-stratified URTI hospitalisations, from 2015 to 2023

| Year | Age groups | | | | | | | |
| --- | --- | --- | --- | --- | --- | --- | --- | --- |
|  | All | <1 | 1 – 4 | 5 – 9 | 10 – 19 | 20 – 59 | ≥60 | ≥65 |
| 2015 | 129 | 22 | 30 | 9 | 5 | 28 | 36 | 31 |
| 2016 | 182 | 29 | 36 | 12 | 9 | 39 | 56 | 49 |
| 2017 | 236 | 31 | 41 | 13 | 10 | 50 | 91 | 82 |
| 2018 | 265 | 32 | 39 | 13 | 9 | 46 | 126 | 107 |
| 2019 | 335 | 32 | 38 | 12 | 9 | 49 | 195 | 156 |
| 2020 | 315 | 13 | 15 | 5 | 9 | 169 | 104 | 82 |
| 2021 | 135 | 16 | 16 | 4 | 4 | 32 | 64 | 53 |
| 2022 | 389 | 28 | 34 | 10 | 7 | 47 | 263 | 221 |
| 2023 | 853 | 35 | 41 | 24 | 14 | 53 | 685 | 569 |

**Supplementary Table 4.** Reported mean weekly age-stratified LRTI hospitalisations, from 2015 to 2023

| Year | Age groups | | | | | | | |
| --- | --- | --- | --- | --- | --- | --- | --- | --- |
|  | All | <1 | 1 – 4 | 5 – 9 | 10 – 19 | 20 – 59 | ≥60 | ≥65 |
| 2015 | 164 | 35 | 52 | 8 | 2 | 16 | 51 | 46 |
| 2016 | 179 | 42 | 62 | 9 | 3 | 18 | 46 | 41 |
| 2017 | 194 | 43 | 66 | 10 | 4 | 20 | 52 | 46 |
| 2018 | 199 | 42 | 73 | 10 | 3 | 20 | 51 | 44 |
| 2019 | 206 | 43 | 74 | 10 | 4 | 21 | 54 | 47 |
| 2020 | 82 | 10 | 24 | 5 | 1 | 14 | 28 | 24 |
| 2021 | 92 | 17 | 46 | 5 | 1 | 7 | 17 | 15 |
| 2022 | 165 | 34 | 80 | 12 | 2 | 11 | 26 | 24 |
| 2023 | 212 | 42 | 72 | 19 | 5 | 19 | 55 | 48 |

**Supplementary Table 5.** Estimated mean yearly age-stratified influenza-associated excess P&I hospitalisation rates (per 100 000 person-years) from 2015-2023. 95% CI in brackets

| Year | Age groups | | | | | | | |
| --- | --- | --- | --- | --- | --- | --- | --- | --- |
|  | All | <1 | 1 – 4 | 5 – 9 | 10 – 19 | 20 – 59 | ≥60 | ≥65 |
| 2015 | 59.9  (48.4-71.6) | 405.3  (320.2-486.0) | 288.1  (193.0-373.4) | 104.2  (77.1-129.8) | 35.5  (22.0-47.2) | 10.7  (4.4-17.5) | 241.2  (182.4-302.3) | 326.2  (243.9-412.8) |
| 2016 | 94.7  (82.3-107.1) | 419.7  (337.1-502.0) | 445.7  (330.9-545.6) | 147.7  (119.5-175.9) | 64.4  (47.4-79.0) | 20.5  (12.7-29.5) | 337.5  (273.6-400.4) | 444.5  (355.1-532.1) |
| 2017 | 111.7  (96.8-126.4) | 610.8  (488.6-722.4) | 527.9  (396.8-638.0) | 130.9  (104.1-154.8) | 52.0  (37.9-63.6) | 22.6  (14.2-32.1) | 401  (331.4-472.9) | 530.7  (436.6-629.3) |
| 2018 | 115.2  (99.2-131.0) | 550.2  (438.2-650.9) | 563.6  (423.6-682.1) | 154.1  (126.8-181.2) | 55.7  (41.2-67.9) | 25.9  (17-35.2) | 388.7  (310.9-464.1) | 510.2  (404.0-611.7) |
| 2019 | 129.8  (110.9-148.4) | 673.3  (540.3-792.6) | 803.0  (612.3-964.7) | 260.4  (214.3-303.1) | 84.9  (62.6-103.6) | 30.0  (20.2-40.9) | 377.5  (294.7-459.5) | 490.7  (378.4-601.7) |
| 2020 | 29.5  (15.2-46.6) | 435.4  (385.7-481.1) | 197.6  (107.1-267.5) | 77.8  (47.7-102.0) | 26.8  (14.1-35.6) | 10.6  (3.8-20.6) | 77.2  (32.4-141.4) | 97.9  (42.1-179.8) |
| 2021 | 10.0  (3.9-20.6) | 85.4  (45.4-142.2) | 26.3  (9.1-46.7) | 6.3  (1.5-10.5) | 4.3  (1.1-7.5) | 3.9  (0.9-9.3) | 53.3  (23.0-97.3) | 66.4  (27.5-125.6) |
| 2022 | 35.2  (10.7-64.2) | 153.8  (55.7-289.5) | 153.6  (29.5-288.1) | 74.3  (41.5-102.4) | 35.0  (23.6-42.3) | 10.5  (3.8-18.7) | 131.2  (29.3-264.0) | 187.2  (42.7-369.9) |
| 2023 | 92.0  (33.9-150.5) | 184.1  (27.3-454.0) | 432.8  (180.8-671.3) | 143  (46.9-244.4) | 62.9  (35.0-85.3) | 27.7  (9.8-45.2) | 258.3  (55.9-497.6) | 294.1  (61.8-597.3) |

**Supplementary Table 6.** Estimated mean yearly age-stratified influenza-associated excess URTI hospitalisation rates (per 100 000 person-years) from 2015-2023. 95% CI in brackets

| Year | Age groups | | | | | | | |
| --- | --- | --- | --- | --- | --- | --- | --- | --- |
|  | All | <1 | 1 – 4 | 5 – 9 | 10 – 19 | 20 – 59 | ≥60 | ≥65 |
| 2015 | 25.0 (18.4-32.0) | 506.3 (303.0-737.4) | 197.5 (122.2-276.1) | 61.0 (42.9-80.0) | 20.4 (14.0-26.6) | 9.1 (6.8-11.2) | 52.4 (39.9-66.5) | 72.9 (54.1-91.7) |
| 2016 | 45.4 (36.3-54.4) | 842.1 (527.2-1158.9) | 282.4 (187.6-376.9) | 113.4 (88.5-137.4) | 43.1 (33.4-52.3) | 15.2 (12.2-18.1) | 94.3 (77.2-112.8) | 126.8 (102.1-152.7) |
| 2017 | 62.6 (49.8-74.5) | 808.7 (471.2-1192.5) | 354.7 (252.6-454.2) | 124.2 (96.6-150.3) | 52.7 (40.7-63.3) | 20.9 (17.0-24.6) | 149.7 (116.7-180.9) | 208.3 (161.6-252.8) |
| 2018 | 67.5 (52.4-81.9) | 911.2 (496.4-1340.3) | 333.4 (220.4-437.1) | 128.2 (99.1-154.0) | 44.1 (32.8-54.1) | 19.0 (15.1-22.6) | 199.4 (155.5-241.5) | 258.2 (200.2-314.1) |
| 2019 | 86.6 (68.1-104.3) | 966.3 (534.1-1412.7) | 333.2 (232.0-436.7) | 110.8 (83.2-137.3) | 51.6 (39.1-61.8) | 19.1 (15.1-23.2) | 292.9 (227.3-354.4) | 357.1 (276.5-432.5) |
| 2020 | 34.2  (9.8-64.9) | 343.4  (138.7-560.0) | 76.9  (25.7-151.6) | 39.3  (18.7-67.7) | 19.0  (9.4-35.5) | 33.2  (5.7-66.8) | 105.8  (51.4-162.5) | 128.7  (69.0-192.4) |
| 2021 | 6.6  (0.7-20.8) | 206.1  (65.0-462.0) | 49.0  (14.8-111.6) | 19.3  (5.0-35.7) | 10.0  (3.5-18.3) | 3.5  (0.8-12.3) | 24.1  (6.6-55.3) | 27.9  (6.8-66.9) |
| 2022 | 4.5  (0.8-28.4) | 195.4  (41.0-698.0) | 29.6  (5.2-123.8) | 61.9  (26.3-99.8) | 16.3  (5.9-32.8) | 5.2  (0.6-15.5) | 27.2  (5.5-160.9) | 478.0  (240.8-701.3) |
| 2023 | 91.2  (32.8-151.3) | 1304.1  (428.2-2176.8) | 222.8  (93.5-369.9) | 239.4  (110.2-336.5) | 62.0  (37.8-82.4) | 13.2  (5.0-22.3) | 550.5  (287.6-805.1) | 646.7  (349.2-933.8) |

**Supplementary Table 7.** Estimated mean yearly age-stratified influenza-associated excess LRTI hospitalisation rates (per 100 000 person-years) from 2015-2023. 95% CI in brackets

| Year | Age groups | | | | | | | |
| --- | --- | --- | --- | --- | --- | --- | --- | --- |
|  | All | <1 | 1 – 4 | 5 – 9 | 10 – 19 | 20 – 59 | ≥60 | ≥65 |
| 2015 | 30.3 (20.7-40.5) | 1409.9 (915.7-1893.0) | 339.2 (224.5-453.8) | 24.5 (13.2-38.7) | 7.6 (5.3-10.0) | 2.4 (1.4-3.9) | 72.8 (51.5-94.6) | 103.9 (72.8-137.3) |
| 2016 | 38.4 (27.7-48.8) | 1826.9 (1182.9-2432.2) | 414.6 (258.5-564.6) | 48.0 (34.8-61.1) | 10.0 (6.6-13.0) | 2.9 (1.4-4.7) | 66.2 (49.7-83.8) | 94.3 (69.4-119.6) |
| 2017 | 44.0 (32.3-55.9) | 1965.1 (1233.6-2619.9) | 444.6 (282.6-613.7) | 42.4 (28.9-57.2) | 13.8 (10.1-17.2) | 4.2 (2.4-6.5) | 77.1 (56.4-98.1) | 108.5 (77.1-139.2) |
| 2018 | 43.6 (30.3-56.1) | 1816.9 (1145.9-2508.3) | 512.5 (312.6-704.1) | 40.1 (23.9-58.8) | 11.7 (8.4-14.8) | 3.5 (1.5-5.9) | 71.9 (51.6-92.3) | 95.8 (67.2-125.2) |
| 2019 | 48.1 (35.2-60.5) | 2196.3 (1456.6-2879.2) | 556.5 (361.4-748.8) | 44.5 (27.1-64.1) | 12.7 (8.3-17.1) | 3.5 (1.6-6.1) | 74.3 (52.8-94.8) | 99.7 (69.5-128.0) |
| 2020 | 10.3  (4.3-16.9) | 309.8  (77.8-546.9) | 105.0  (34.4-205.9) | 27.3  (8.3-51.6) | 5.5  (2.6-9.0) | 1.3  (0.3-3.3) | 28.1  (16.6-40.6) | 33.0  (18.8-48.6) |
| 2021 | 5.5  (2.0-11.1) | 267.1  (76.7-560.3) | 105.4  (24.2-230.0) | 20.4  (8.1-37.1) | 4.1  (1.4-6.7) | 1.7  (0.8-2.8) | 6.2  (1.3-16.0) | 8.9  (1.5-20.8) |
| 2022 | 8.2  (0.9-24.8) | 323.6  (13.9-1066.8) | 43.2  (6.7-137.3) | 32.5  (2.8-89.5) | 4.5  (0.6-13.5) | 2.3  (0.4-4.7) | 27.7  (9.9-48.6) | 39.7  (14.2-66.8) |
| 2023 | 20.4  (10.2-31.8) | 702.6  (158.9-1501.5) | 245.7  (73.2-473.6) | 34.9  (0.0-119.0) | 8.5  (0.3-22.9) | 3.9  (0.4-10.3) | 43.2  (17.3-71.5) | 50.8  (18.9-86.8) |

**Supplementary Table 8.** Estimated yearly age-stratified influenza-associated excess P&I, URTI and LRTI hospitalisation proportion from 2015-2023. 95% CI in brackets

| Year | Age groups | | | | | | | |
| --- | --- | --- | --- | --- | --- | --- | --- | --- |
|  | All | <1 | 1 – 4 | 5 – 9 | 10 – 19 | 20 – 59 | ≥60 | ≥65 |
| 2015 | 19.0  (14.4-23.7) | 28.2  (18.7-37.8) | 27.1  (17.7-36.2) | 26.7  (18.7-34.9) | 37.3  (24.2-49.2) | 15.1  (8.5-22.1) | 17.2  (12.9-21.8) | 17.3  (12.8-22.1) |
| 2016 | 24.4  (20.0-28.8) | 31.2  (20.7-41.3) | 31.3  (21.3-40.7) | 35.1  (27.6-42.6) | 46.7  (34.7-57.3) | 20.5  (13.9-27.7) | 20.9  (16.8-25.1) | 20.6  (16.3-24.9) |
| 2017 | 26.0  (21.3-30.6) | 30.6  (19.8-40.9) | 33.0  (23.2-42.4) | 34.7  (26.8-42.3) | 47.7  (35.7-58.0) | 22.1  (15.5-29.3) | 22.8  (18.4-27.4) | 22.7  (18.1-27.4) |
| 2018 | 26.0  (20.9-30.9) | 29.5  (18.7-40.5) | 33.0  (22.4-42.7) | 36.0  (27.9-44.0) | 47.4  (35.0-58.2) | 23.0  (16.0-30.3) | 23.4  (18.4-28.3) | 23.3  (18.1-28.3) |
| 2019 | 26.6  (21.6-31.5) | 33.0  (21.7-43.7) | 35.8  (25.5-45.5) | 36.8  (28.8-44.7) | 48.9  (36.0-59.8) | 22.5  (15.7-30.0) | 23.3  (18.0-28.5) | 23.4  (17.9-28.7) |
| 2020 | 9.9  (3.9-17.1) | 28.7  (15.8-41.8) | 23.6  (10.4-38.9) | 36.0  (18.7-55.2) | 30.1  (15.3-47.1) | 11.6  (2.5-23.3) | 9.5  (4.5-15.6) | 9.3  (4.6-15.0) |
| 2021 | 4.2  (1.3-10.0) | 11.3  (3.8-23.5) | 9.2  (25-19.8) | 24.0  (7.6-43.4) | 27.4  (9.0-48.2) | 7.2  (2.0-19.1) | 4.6  (1.7-9.4) | 4.5  (1.6-9.2) |
| 2022 | 5.1  (1.3-12.5) | 7.2  (1.2-21.9) | 5.5  (1.0-13.3) | 26.3  (11.0-45.6) | 35.1  (18.9-55.7) | 11.5  (3.1-24.9) | 5.6  (1.4-14.3) | 16.9  (7.1-27.2) |
| 2023 | 13.2  (5.0-21.6) | 17.1  (4.8-32.3) | 18.7  (7.2-31.4) | 27.3  (10.3-45.8) | 37.1  (20.4-53.1) | 20.1  (6.8-34.9) | 14.6  (6.2-23.5) | 13.9  (6.0-22.7) |

**Supplementary Table 9.** Estimated mean yearly age-stratified RSV-associated excess P&I hospitalisation rates (per 100 000 person-years) from 2015-2023. 95% CI in brackets

| Year | Age groups | | | | | | | |
| --- | --- | --- | --- | --- | --- | --- | --- | --- |
|  | All | <1 | 1 – 4 | 5 – 9 | 10 – 19 | 20 – 59 | ≥60 | ≥65 |
| 2015 | 17.3  (10.8-26.4) | 200.4  (124.0-281.1) | 80.1  (34.4-127.6) | 46.7  (27.2-68.9) | 12.2  (6.5-19.9) | 5.0  (2.7-8.1) | 103.7  (66.1-149.5) | 141.9  (92.2-207.2) |
| 2016 | 22.8  (14.9-32.2) | 171.2  (114.0-236.6) | 99.1  (56.7-156.1) | 47.2  (28.7-70.8) | 19.6  (11.5-29.2) | 6.5  (3.8-10.2) | 109.1  (71.6-161.3) | 147.6  (96.5-214.7) |
| 2017 | 31.3  (21.3-43.5) | 251.5  (158.4-351.4) | 126.9  (69.6-190.4) | 39.7  (22.2-63.2) | 15.1  (8.6-23.2) | 6.4  (3.7-10.3) | 160.1  (113.9-216.4) | 210.1  (151.8-289.5) |
| 2018 | 26.6  (16.8-39.8) | 201.7  (134.7-285.7) | 134.7  (85.4-203.0) | 47.5  (26.9-71.9) | 21.2  (13.2-29.7) | 7.6  (4.0-12.3) | 118.3  (70.4-178.6) | 158.3  (92.1-239.0) |
| 2019 | 31.4  (18.9-47.8) | 247.7  (133.5-372.5) | 195.3  (104.7-297.6) | 92.5  (51.7-138.7) | 29.2  (16.1-43.3) | 10.0  (5.5-15.5) | 110.8  (60.1-177.7) | 144.5  (76.9-236.7) |
| 2020 | 12.8  (5.2-27.7) | 97.1  (32.4-203.4) | 69.6  (18.3-147.1) | 14.6  (3.5-43.0) | 13.2  (3.3-25.5) | 8.8  (3.3-16.5) | 54.9  (19.0-114.7) | 72.8  (27.7-148.2) |
| 2021 | 12.5  (4.1-26.4) | 107.7  (62.6-163.3) | 49.7  (19.1-81.0) | 5.2  (0.9-10.3) | 5.9  (1.9-8.7) | 4.4  (0.8-11.1) | 66.1  (28.9-121.2) | 87.3  (37.7-161.4) |
| 2022 | 7.9  (0.8-26.2) | 177.7  (62.0-303.6) | 39.4  (1.3-144.8) | 20  (2.9-44.6) | 6.3  (1.2-15.8) | 2.2  (0.6-6.9) | 55.5  (4.8-162.3) | 76.9  (7.2-218.9) |
| 2023 | 36.8  (10.1-78.5) | 275.6  (57.9-548.7) | 183.1  (27.3-418.5) | 41.6  (6.9-137.8) | 7.4  (0.0-32.3) | 5.1  (0.9-15.8) | 133.6  (27.1-297.2) | 178  (39.6-398.5) |

**Supplementary Table 10.** Estimated mean yearly age-stratified RSV-associated excess URTI hospitalisation rates (per 100 000 person-years) from 2015-2023. 95% CI in brackets

| Year | Age groups | | | | | | | |
| --- | --- | --- | --- | --- | --- | --- | --- | --- |
|  | All | <1 | 1 – 4 | 5 – 9 | 10 – 19 | 20 – 59 | ≥60 | ≥65 |
| 2015 | 7.2 (4.2-11.2) | 317.0 (212.9-431.7) | 62.2 (33.7 - 96.7) | 26.7 (15.2 - 39.2) | 8.0 (4.3 - 12.3) | 3.6 (2.0 - 5.4) | 20.8 (12.2 - 31.5) | 29.7 (18.1 - 45.2) |
| 2016 | 10.8 (6.2-16.1) | 450.2 (297.8-628.9) | 82.1 (53.1 - 118.5) | 42.2 (27.5 - 58.9) | 16.1 (10.6 - 22.6) | 4.3 (2.5 - 6.6) | 29.3 (18.2 - 43.7) | 39.4 (22.9 - 59.8) |
| 2017 | 13.9 (8.9-21.4) | 400.2 (253.3-580.3) | 105.9 (70.4 - 151.1) | 38.7 (25.1 - 57.1) | 17.7 (10.3 - 25.8) | 6.3 (4.2 - 9.3) | 36.6 (19.3 - 60.2) | 50.9 (24.7 - 85.5) |
| 2018 | 10.8 (5.9-17.6) | 462.3 (270.5-684.4) | 101.1 (63.4 - 143.9) | 36.6 (19.4 - 57.0) | 14.6 (8.5 - 21.4) | 4.7 (2.8 - 7.2) | 41.9 (23 - 69.2) | 55.5 (30.6 - 90.9) |
| 2019 | 17.9 (10.5-28.7) | 484.4 (292.0-730.3) | 104.7 (64.1 - 158.3) | 33.7 (17.8 - 54.4) | 18.3 (9.8 - 27.5) | 4.7 (2.8 - 7.6) | 58.3 (33.2 - 99.3) | 77.8 (41.5 - 133.4) |
| 2020 | 32.2  (11.2-59.9) | 275.7  (129.8-475.6) | 57.5  (24.4-120.7) | 33.3  (16.8-57.2) | 23.7  (11.5-40.1) | 35.5  (6.2-70.2) | 50.7  (14.9-108.2) | 55.2  (17.5-121.7) |
| 2021 | 13.1  (1.7-31.5) | 391.8  (97.3-723.9) | 85.0  (22.0-161.5) | 18.1  (2.5-37.3) | 11.8  (4.0-21.5) | 7.8  (2.1-17.5) | 35.2  (8.8-75.7 | 38.6  (8.4-88.2) |
| 2022 | 22.8  (5.8-50.0) | 364.3  (61.7-905.0) | 81.4  (9.7-211.8) | 11.1  (1.0-35.3) | 7.6  (1.9-17.5) | 3.8  (0.4-12.1) | 112.6  (27.2-251.9) | 72.9  (14.6-243.8) |
| 2023 | 14.3  (0.2-85.9) | 245.2  (17.7-925.6) | 155.9  (37.2-308.8) | 92.5  (22.0-174.6) | 45.4  (20.3-70.9) | 2.8  (0.0-15.0) | 90.8  (3.7-470.5) | 79.4  (1.1-480.3) |

**Supplementary Table 11.** Estimated mean yearly age-stratified RSV-associated excess LRTI hospitalisation rates (per 100 000 person-years) from 2015-2023. 95% CI in brackets

| Year | Age groups | | | | | | | |
| --- | --- | --- | --- | --- | --- | --- | --- | --- |
|  | All | <1 | 1 – 4 | 5 – 9 | 10 – 19 | 20 – 59 | ≥60 | ≥65 |
| 2015 | 11.4 (7.5-16.2) | 691.2 (422.3-980.9) | 155.0 (100.3-219.3) | 18.5 (10.4-30.1) | 5.8 (3.9-7.8) | 2.5 (1.8-3.3) | 32.4 (19.9-47.4) | 44.4 (26.3-65.2) |
| 2016 | 10.4 (6.3-15.6) | 847.8 (561.5-1185.4) | 139.3 (86.1-212.5) | 37.4 (27.2-48.1) | 6.6 (4.8-8.9) | 2.4 (1.7-3.2) | 22.0 (13.5-33.1) | 29.8 (19.1-44.5) |
| 2017 | 13.7 (8.5-19.8) | 888.2 (549.7-1271.2) | 144.9 (85.0-228.6) | 31.6 (21.0-43.2) | 9.3 (6.7-12.5) | 3.7 (2.7-4.8) | 25.7 (14.8-39.6) | 34.8 (20.3-52.9) |
| 2018 | 14.6 (9.4-20.7) | 1090.6 (796.7-1425.4) | 213.2 (133.6-309.2) | 27.5 (17.5-41.5) | 7.4 (5.2-10.0) | 2.3 (1.5-3.4) | 19.8 (10.7-33.5) | 24.5 (13.5-41.6) |
| 2019 | 17.7 (12.2-24.3) | 1294.8 (923.1-1711.2) | 235.4 (162.1-336.2) | 33.9 (20.3-49.2) | 8.8 (5.7-12.1) | 2.9 (1.7-4.4) | 21.3 (10.9-36.6) | 27.1 (13.9-46.3) |
| 2020 | 6.2  (1.4-12.4) | 283.9  (99.1-487.8) | 67.7  (25.7-145.4) | 23.1  (7.3-45.3) | 5.8  (2.9-9.1) | 1.6  (0.3-3.9) | 22.8  (10.4-35.7) | 30.5  (15.4-46.3) |
| 2021 | 14.2  (6.9-22.5) | 891.8  (47.9-1250.6) | 209.2  (58.2-380.8) | 15.4  (4.5-34.8) | 4.3  (1.5-6.9) | 1.8  (0.7-3.0) | 13.0  (4.4-24.5) | 19.5  (7.6-32.8) |
| 2022 | 2.0  (0.4-9.9) | 231.1  (12.0-862.5) | 37.3  (9.6-277.5) | 35.7  (2.2-84.9) | 7.2  (0.8-14.4) | 1.7  (0.3-3.8) | 8.4  (2.1-23.0) | 11.8  (2.9-31.9) |
| 2023 | 11.2  (3.5-22.5) | 999.5  (272.0-1858.8) | 307.9  (39.4-658.7) | 58.1  (5.8-135.5) | 16.3  (3.9-28.6) | 3.8  (0.6-8.6) | 1.9  (0.0-29.7) | 10.6  (1.0-48.0) |

**Supplementary Table 12.** Estimated yearly age-stratified RSV-associated excess P&I, URTI and LRTI hospitalisation proportion from 2015-2023. 95% CI in brackets

| Year | Age groups | | | | | | | |
| --- | --- | --- | --- | --- | --- | --- | --- | --- |
|  | All | <1 | 1 – 4 | 5 – 9 | 10 – 19 | 20 – 59 | ≥60 | ≥65 |
| 2015 | 5.9  (3.7-8.9) | 14.7  (9.2-20.6) | 9.8  (5.5-14.6) | 12.9  (7.4-19.4) | 15.3  (8.7-23.5) | 7.5  (4.4-11.4) | 7.4  (4.6-10.7) | 7.4  (4.7-10.9) |
| 2016 | 6.0  (3.8-8.7) | 14.8  (9.8-20.7) | 8.8  (5.4-13.3) | 14.4  (9.5-20.2) | 16.8  (10.7-24.1) | 7.0  (4.2-10.6) | 6.7  (4.3-10.0) | 6.7  (4.3-9.9) |
| 2017 | 7.0  (4.6-10.1) | 13.9  (8.7-19.9) | 9.4  (5.6-14.2) | 12.8  (8.0-19.1) | 16.9  (10.3-24.7) | 7.6  (4.9-11.3) | 8.1  (5.4-11.5) | 7.9  (5.3-11.5) |
| 2018 | 6.0  (3.7-9.0) | 15.8  (10.8-21.6) | 10.5  (6.6-15.4) | 12.5  (7.1-19.0) | 18.4  (11.4-26.0) | 6.9  (4.0-10.9) | 6.4  (3.7-10.0) | 6.4  (3.7-10.0) |
| 2019 | 6.7  (4.2-10.2) | 17.4  (11.6-24.2) | 11.3  (7.0-16.7) | 14.2  (8.0-21.5) | 18.5  (10.4-27.2) | 7.5  (4.3-11.7) | 6.0  (3.3-9.8) | 6.2  (3.3-10.3) |
| 2020 | 6.8  (2.4-13.4) | 17.3  (6.9-30.7) | 12.1  (4.3-25.7) | 17.7  (6.9-36.3) | 25.0  (10.4-43.9) | 11.8  (2.5-23.3) | 5.8  (2.0-11.7) | 5.7  (2.2-11.3) |
| 2021 | 7.6  (2.4-15.4) | 28.1  (12.9-43.1) | 17.5  (5.1-31.8) | 20.2  (4.1-43.0) | 32.6  (11.0-55.0) | 11.0  (2.9-24.9) | 6.4  (2.3-12.3) | 6.3  (2.3-12.2) |
| 2022 | 3.5  (0.7-9.1) | 8.3  (1.4-22.1) | 3.8  (00.5-15.4) | 10.4  (1.0-25.7) | 13.3  (2.5-30.0) | 4.9  (0.9-14.5) | 5.3  (1.0-13.2) | 3.9  (0.6-11.8) |
| 2023 | 4.0  (0.9-12.1) | 11.9  (2.7-26.0) | 13.4  (2.2-28.7) | 12.6  (2.3-29.3) | 19.2  (6.7-36.7) | 5.3  (0.7-17.7) | 3.9  (0.5-13.6) | 3.8  (0.6-13.0) |

**Supplementary Table 13.** Estimated mean yearly age-stratified SARS-CoV-2-associated excess P&I hospitalisation rates (per 100 000 person-years) from 2020-2023. 95% CI in brackets

| Year | Age groups | | | | | | | |
| --- | --- | --- | --- | --- | --- | --- | --- | --- |
|  | All | <1 | 1 – 4 | 5 – 9 | 10 – 19 | 20 – 59 | ≥60 | ≥65 |
| 2020 | 21.2  (11.0-37.5) | 72.1  (22.0-163.1) | 35.9  (13.2-106.2) | 18.9  (4.3-44.6) | 7.9  (2.3-19.2) | 13.8  (7.0-22.6) | 74.9  (39.2-139.1) | 97.6  (53.3-180.9) |
| 2021 | 54.3  (43.8-66.4) | 81.0  (45.4-123.3) | 27.9  (8.9-49.8) | 7.0  (1.6—11.0) | 4.3  (1.0-7.6) | 16.7  (11.7-22.5) | 239.4  (198.6-289.1) | 301.6  (248.6-368.1) |
| 2022 | 83.7  (53.6-113.9) | 84.4  (18.6-202.8) | 7.6  (0.7-88.1) | 33.0  (8.5-62.5) | 12.2  (3.4-23.9) | 8.7  (2.8-15.6) | 409.7  (275.2-543.6) | 525.2  (341.9-707.6) |
| 2023 | 95.4  (60.2-132.5) | 215.4  (66.9-420.3) | 80.5  (21.8-256.1) | 87.5  (22.7-174.2) | 5.5  (1.2-21.7) | 13.5  (6.5-22.3) | 422.8  (286.6-562.4) | 582.6  (392.6-777.3) |

**Supplementary Table 14.** Estimated mean yearly age-stratified SARS-CoV-2-associated excess URTI hospitalisation rates (per 100 000 person-years) from 2020-2023. 95% CI in brackets

| Year | Age groups | | | | | | | |
| --- | --- | --- | --- | --- | --- | --- | --- | --- |
|  | All | <1 | 1 – 4 | 5 – 9 | 10 – 19 | 20 – 59 | ≥60 | ≥65 |
| 2020 | 93.0  (72.3-118.8) | 235.2  (112.3-431.1) | 59.7  (24.1-120.1) | 33.3  (17.3-56.2) | 22.0  (10.9-37.9) | 115.2  (86.9-141.8) | 61.9  (23.4-121.1) | 75.4  (32.8-144.9) |
| 2021 | 47.4  (36.6-58.8) | 176.5  (54.0-428.3) | 38.6  (11.9-103.6) | 14.1  (3.4-32.3) | 8.7  (2.1-17.2) | 22.5  (16.8-27.7) | 99.5  (64.6-133.1) | 127.0  (87.6-166.8) |
| 2022 | 105.6  (73.8-135.3) | 406.9  (65.5-969.9) | 37.6  (5.6-157.3) | 24.8  (4.7-68.2) | 22.4  (8.8-37.0) | 14.9  (4.5-24.9) | 532.8  (380.6-666.2) | 748.7  (595.1-891.1) |
| 2023 | 191.5  (147.1-234.2) | 806.7  (307.9-1412.5) | 95.8  (25.2-204.2) | 72.7  (17.2-147.9) | 24.7  (8.7-44.3) | 28.5  (18.3-37.4) | 1073.0  (832.9-1295.3) | 1302.9  (1036.7-1551.9) |

**Supplementary Table 15.** Estimated mean yearly age-stratified SARS-CoV-2-associated excess LRTI hospitalisation rates (per 100 000 person-years) from 2020-2023. 95% CI in brackets

| Year | Age groups | | | | | | | |
| --- | --- | --- | --- | --- | --- | --- | --- | --- |
|  | All | <1 | 1 – 4 | 5 – 9 | 10 – 19 | 20 – 59 | ≥60 | ≥65 |
| 2020 | 3.6  (1.0-9.3) | 143.4  (31.1-352.8) | 72.8  (26.2-146.6) | 25.8  (7.9-46.0) | 5.1  (2.5-8.2) | 1.8  (0.5-4.1) | 12.9  (4.9-25.8) | 17.7  (7.5-32.3) |
| 2021 | 4.2  (1.5-8.9) | 225.3  (68.5-552.6) | 74.4  (16.4-170.8) | 13.6  (3.2-30.0) | 3.3  (1.1-5.8) | 1.9  (1.0-3.0) | 8.0  (2.0-18.1) | 10.0  (2.2-22.6) |
| 2022 | 2.0  (0.5-12.9) | 96.3  (9.6-784.6) | 54.3  (7.3-331.0) | 18.6  (1.1-68.1) | 5.9  (0.4-13.9) | 2.0  (0.4-4.4) | 27.9  (10.1-46.1) | 36.6  (13.7-61.0) |
| 2023 | 11.3  (5.6-19.4) | 568.6  (153.0-1225.0) | 144.1  (20.7-373.2) | 29.0  (0.4-89.1) | 4.5  (0.8-12.5) | 4.2  (1.0-8.0) | 46.4  (25.2-68.6) | 62.1  (35.5-89.0) |

**Supplementary Table 16.** Estimated yearly age-stratified SARS-CoV-2-associated excess P&I, URTI and LRTI hospitalisation proportion from 2020-2023. 95% CI in brackets

| Year | Age groups | | | | | | | |
| --- | --- | --- | --- | --- | --- | --- | --- | --- |
|  | All | <1 | 1 – 4 | 5 – 9 | 10 – 19 | 20 – 59 | ≥60 | ≥65 |
| 2020 | 15.7  (11.3-22.1) | 11.9  (4.4-24.9) | 10.5  (4.0-23.2) | 19.5  (7.4-36.6) | 20.5  (9.2-38.3) | 33.6  (24.3-43.3) | 6.8  (3.1-12.9) | 6.8  (3.3-12.8) |
| 2021 | 20.3  (15.7-25.7) | 9.7  (3.4-22.3) | 7.2  (1.9-16.5) | 18.1  (4.3-38.2) | 24.2  (6.3-45.4) | 32.3  (23.2-41.8) | 19.3  (14.7-24.5) | 19.0  (14.7-24.2) |
| 2022 | 20.3  (13.6-27.8) | 6.3  (1.0-20.9) | 2.4  (0.3-14.0) | 11.9  (2.2-31.0) | 25.5  (8.0-47.0) | 16.4  (4.9-28.7) | 29.3  (20.1-37.9) | 31.4  (22.8-39.7) |
| 2023 | 19.3  (13.8-25.0) | 12.4  (4.1-23.9) | 6.6  (1.4-17.3) | 12.4  (2.6-26.9) | 9.7  (3.0-21.9) | 20.7  (11.5-30.4) | 26.4  (19.6-32.9) | 27.3  (20.5-33.9) |
